# Examining the Transmission of Visible Light through Electrospun Nanofibrous PCL Scaffolds for Corneal Tissue Engineering

**DOI:** 10.3390/nano11123191

**Published:** 2021-11-25

**Authors:** Marcus Himmler, Dirk W. Schubert, Thomas A. Fuchsluger

**Affiliations:** 1 Department of Ophthalmology, University Medical Center Rostock, Doberaner Straße 140, 18057 Rostock, Germany; 2 Institute of Polymer Materials, Friedrich-Alexander University Erlangen-Nuremberg, Martenstraße 7, 91058 Erlangen, Germany; dirk.schubert@fau.de

**Keywords:** electrospinning, tissue engineering, cornea, transparency, nanofibers, polycaprolactone

## Abstract

The transparency of nanofibrous scaffolds is of highest interest for potential applications like corneal wound dressings in corneal tissue engineering. In this study, we provide a detailed analysis of light transmission through electrospun polycaprolactone (PCL) scaffolds. PCL scaffolds were produced via electrospinning, with fiber diameters in the range from (35 ± 13) nm to (167 ± 35) nm. Light transmission measurements were conducted using UV–vis spectroscopy in the range of visible light and analyzed with respect to the influence of scaffold thickness, fiber diameter, and surrounding medium. Contour plots were compiled for a straightforward access to light transmission values for arbitrary scaffold thicknesses. Depending on the fiber diameter, transmission values between 15% and 75% were observed for scaffold thicknesses of 10 µm. With a decreasing fiber diameter, light transmission could be improved, as well as with matching refractive indices of fiber material and medium. For corneal tissue engineering, scaffolds should be designed as thin as possible and fabricated from polymers with a matching refractive index to that of the human cornea. Concerning fiber diameter, smaller fiber diameters should be favored for maximizing graft transparency. Finally, a novel, semi-empirical formulation of light transmission through nanofibrous scaffolds is presented.

## 1. Introduction

In the field of tissue engineering, electrospun scaffolds are commonly used [1]; however, optical properties are in general of minor importance in most applications. In the case of tissue engineering for ophthalmic applications, the transparency of the graft is of highest interest. The cornea is the window of the eye, and its transparency is essential for human beings. Recently, electrospun scaffolds have been discussed for use in ophthalmic applications such as wound dressings after corneal surgery [2,3,4,5,6] or as artificial DMEK (Descemet Membrane Endothelial Keratoplasty) grafts [7,8] for treating patients with corneal endothelial cell pathologies. In both cases, the transparency of the scaffold is of major importance for patients’ immediate benefit after surgery. The transparency of a healthy cornea, which is the reference material in this case, is 85–99% in the visible spectrum [9]; hence, a similar transparency is sought for artificial grafts.

For corneal tissue engineering, additionally to xenogeneic tissue like decellularized corneas [10,11,12], different materials and approaches have been investigated, including nanofibers [5,7,13], hydrogels [4,14,15], and composites thereof [16,17,18]. The transparency of the investigated materials was usually determined by light transmission measurements of individual samples with discrete scaffold thicknesses, and no general transmission study was conducted [6,7,13,17]. The comparison of individual scaffolds with different specifications, such as material or fiber diameter, always presents the problem of insufficient accuracy in scaffold thickness. Beside the field of biomaterials, the optical properties of nanofiber scaffolds were mostly investigated for optoelectronic and energy-related developments to enhance their efficiency [19,20,21].

PCL is a well-studied material in the field of tissue engineering, in particular in the field of corneal tissue engineering [22]. Although known for its opacity, it seems worthwhile to further study PCL due to its remarkable properties, as it is biodegradable and easy to blend with other polymers and has good mechanical strength.

So far, only a few studies were conducted on the transparency of PCL nanofiber scaffolds. For example, Park et al. [23] measured light transmission through electrospun PCL scaffolds using two different fiber diameters and wavelengths. Using an integrating sphere, Park et al. were able to measure directly transmitted and reflected fractions of the incident beam. Their observations indicated that the scattering of the nanofibrous structure is the dominant factor, compared to the light absorption by the material. However, only two discrete wavelengths were investigated, and the influence of a surrounding medium was neglected.

From a physical point of view, the transmission of an electromagnetic wave through a medium can be defined as

(1) $$T=\frac{I}{I_{0}}$$

and describes the transparency of a material. The incremental decrease in light intensity dI within an infinitesimal distance dx is proportional to the incident beam I

(2) dI=−µI dx

which can be simply integrated to

(3) $$T=\frac{I}{I_{0}}=\exp(-µx)$$

for I = I_0_ at x = 0. The parameter µ, known as the extinction coefficient, describes the absorption and scattering of the electromagnetic wave within the volume and can be written as

(4) $${µ}_{\mathrm{total}}{=µ}_{\mathrm{absorption}}{\;+\;µ}_{\mathrm{scattering}}$$

**Figure 1:**
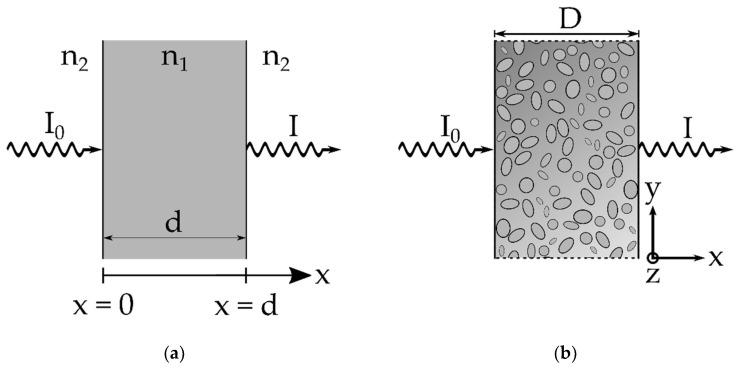
Schematic of an incident beam with intensity I_0_ in the x direction passing through (**a**) a homogenous volume of thickness d with the optical interfaces at the n_2_/n_1_ and n_1_/n_2_ transitions and (**b**) a planar scaffold in the y–z-plane of thickness D, consisting of single nanofibers with fiber diameter d. Propagation of the incident beam in the x direction.

Additionally, the incident electromagnetic wave can be reflected at the interface between two optical adjacent phases, characterized by their refractive indices n_i_. If vertical incidence and polarization of the light are of no relevance, the Fresnel equation [24,25] yields the reflectance R, reducing the light transmission T to

(5) $$T_{\mathrm{reflection}}=1\;-\;R=1\;-\;\frac{{\left(n_{1}-n_{2}\right)}^{2}}{{\left(n_{1}+n_{2}\right)}^{2}}$$

where n_1_ and n_2_ are the refractive indices of the surrounding medium and the material, according to Figure 1a. When an electromagnetic wave passes through a volume, reflectance occurs at the n_1_/n_2_ as well as at the n_2_/n_1_ interfaces. Thus, combining Equations (3)–(5) and neglecting multibeam interference, the overall light transmission through a homogenous volume of thickness d can be written as

(6) $$T=\frac{I}{I_{0}}{=T}_{\mathrm{reflection}}^{2}\exp\left(-\left({µ}_{\mathrm{absorption}}{\;+\;µ}_{\mathrm{scattering}}\right)d\right)$$

In the case of a nanofibrous scaffold of thickness D consisting of nanofibers with a fiber diameter d, as displayed in Figure 1b, µ_absorption_ describes light absorption within each fiber, and µ_scattering_ describes light scattering at the individual fibers. The scattering coefficient µ_scattering_ depends on the scattering cross section of the scatterers, i.e., the nanofibers. The scattering cross section for thin fibers was firstly described by Rayleigh in 1881 [26], and a detailed derivation can be found in [27]. The wavelength-dependent total scattering cross section per unit length of a single isolated fiber of random orientation, with its fiber axis in the y–z-plane and an incident beam perpendicular to the fiber axis and therefore normal to the y–z-plane, is given by

(7) $$\sigma_{\mathrm{scattering}}(\lambda )=\frac{n_{1}^{3}\pi^{3}{\left({\pi r}^{2}\right)}^{2}{\left(m^{2}\;-\;1\right)}^{2}}{\lambda^{3}}\left(1+\frac{2}{\;{{(m}^{2}+1)}^{2}}\right)$$

where r is the fiber radius, λ is the wavelength, and m is the ratio between the refractive indices n_1_ (fiber) and n_2_ (medium). Derived from the dielectric needle approximation, Equation (7) has been used extensively to describe the natural transparency of the mammalian cornea [28,29,30,31,32]. For a porous scaffold, which is the case for electrospun scaffolds, reduction in light transmission occurs for every interaction with individual fibers. The total light transmission through a nanofibrous scaffold should therefore be describable through the scaffold’s thickness, the diameter of the nanofibers, and the refractive indices of the fiber material and the surrounding medium.

For an application-oriented field of research, such as corneal tissue engineering, a general equation, describing the influencing parameters of nanofibrous scaffold transparency, is substantial. Therefore, in this study, electrospun PCL nanofibrous scaffolds with different fiber diameters were investigated regarding their optical properties. Using UV–vis spectroscopy measurements, light transmission through the scaffolds was analyzed with regard to scaffold thickness, fiber diameter, and surrounding medium. Using statistical modelling, power laws were derived for an appropriate description of the data within the experimental error. Finally, design principles were formulated from the experimental findings to promote further research in the field of corneal tissue engineering.

## 2. Materials and Methods

Polycaprolactone (PCL) nanofiber scaffolds were produced via electrospinning. The method is well described in the literature (e.g., [33]; a theoretical description can be found in [34]). In brief, a polymer melt or polymer solution is extruded through a needle. The polymer solution is stretched due to the electrical forces in the electric field, which is set between the needle and a grounded collector. By varying the polymer concentration, different fiber diameters can be fabricated. The spinning solution was prepared from PCL (M_W_ = 80,000 g mol^−1^, Sigma Aldrich, Saint Louis, MO, USA) dissolved in a 7:3 mixture of formic acid and acetic acid (both Carl Roth GmbH + Co. KG, Karlsruhe, Germany). Fiber diameter was evaluated using SEM images (CrossBeam Carl Zeiss Microscopy GmbH, Oberkochen, Germany) and ImageJ software. In preliminary experiments, for each solution, a working window was identified, focusing on a homogenous fiber morphology and sufficient fiber yield. The electrospinning parameters as well as the resulting mean fiber diameters ± standard deviation are given in Table 1.

With increasing spinning time, the scaffold thickness could be adjusted. Due to the similar flow rates, increasing spinning concentrations and thus fiber diameter led to a reduced spinning time for the desired scaffold thicknesses. Scaffolds were fabricated with a desired thickness from 1 µm to 50 µm. Within this range, application-oriented conclusions towards predicting light transmission through the nanofibrous scaffolds could be drawn.

**Figure 2:**
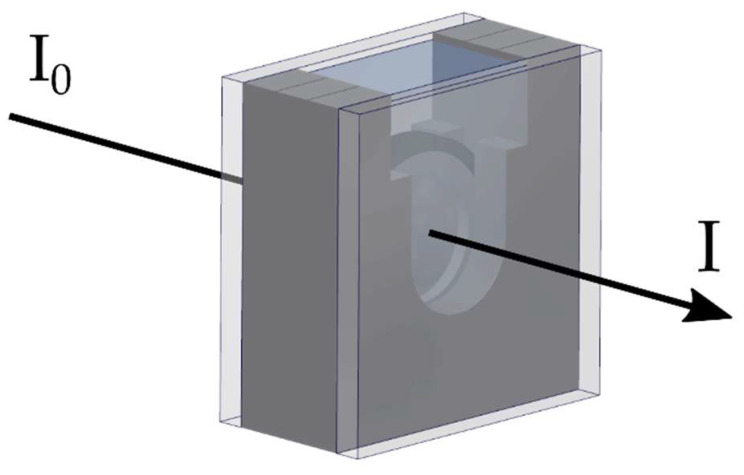
Schematic of the incident and transmitted light intensity through the cuvette. The incident light falls perpendicular to the plane of the scaffold and thus perpendicular to the fiber axis. The cuvette was filled with either ethanol (EtOH) or phosphate-buffered saline (PBS).

Scaffolds were fixed in tissue carrier rings (9 mm inner diameter, Minucells and Minutissue, Bad Abbach, Germany), and the thickness of each scaffold was measured using a digital contact sensor (GT series, Keyence, Itasca, IL, USA). Therefore, the scaffolds, fixed in the tissue carrier rings, were sandwiched between a cylindrical base (8 mm in diameter) and a circular glass platelet (4.5 mm in diameter). Subsequently, the net thickness was measured over a scaffold area of approximately 16 mm^2^. For the measurement of light transmission through the scaffolds, a UV–vis spectrometer (Specord 210 plus, Analytik Jena GmbH, Jena, Germany) was used. Therefore, the scaffolds were placed in a cuvette of personal proprietary (e.g., Figure 2), ensuring that the scaffolds were kept in place perpendicular to the incident, monochromatic beam. The cuvette was filled with either ethanol (EtOH) (Carl Roth GmbH + Co. KG, Karlsruhe, Germany) or phosphate-buffered saline (PBS) (VWR International GmbH, Darmstadt, Germany) to investigate the influence of different surrounding media. Light transmission measurements were conducted from 380 nm to 780 nm with an increment of one nanometer. Prior to every measurement, a calibration scan was performed to normalize the measured intensity to the experimental set-up, consequently I_0_ (λ) = 100%. For each scaffold type, at least 50 scaffolds were measured, resulting in over 250,000 individual wavelength–transmission data points.

**Figure 3:**
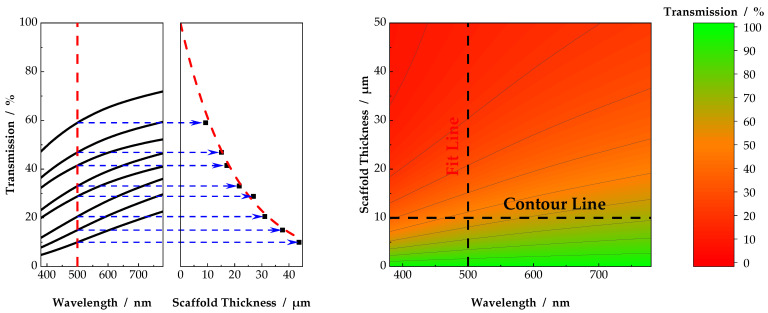
From the individual transmission values, transmission-versus-scaffold thickness plots were generated for discrete wavelengths. Using the fit lines, contour plots were generated for individual scaffolds and enclosing media. Using the contour lines, a comparison between different scaffolds and environmental parameters at various scaffold thicknesses could be made.

From the individual wavelength–transmission data, discrete thickness–transmission data for defined wavelengths were plotted, as shown in Figure 3. Starting from 380 nm, with an increment of 10 nm, fit lines were plotted using an exponential decay function

(8) $$T_{\mathrm{fit}}{=(100\;-\;T}_{\mathrm{background}})\;\exp\left(-\mathrm{mx}\right){+T}_{\mathrm{background}}$$

where T_background_ accounts for the diffuse light transmission of thick scaffolds >50 µm, where the measured light transmission is usually in the range of a few percent. Utilizing Equation (8), an optimal description of the data in the thickness range of interest was reached. Data fitting was performed as a two-stage process using Origin 2019 (OriginLab Corporation, Northampton, MA, USA). After the first fitting, data points with an individual residuum higher than 1.5 times the externally studentized residuum of the fit function were removed from the dataset, and fitting was then repeated with the processed dataset. Usually, outliers originated from false thickness measurements, due to the thickness measurement in contact mode or to an inhomogeneous thickness distribution of the scaffolds. Finally, fit lines were combined in a contour plot, with the wavelength on the x-axis, the scaffold thickness on the y-axis, and the light transmission as colored grading. Between the fit lines, a linear interpolation was presumed. With this approach, errors in the determination of the scaffold thickness or light transmission could be eliminated by averaging a large amount of data. From the contour plot, contour lines of arbitrary thickness can be extracted for an exact comparison of different experimental groups. To give an estimation of the experimental error, contour lines are presented with error bars indicating the 95% confidence interval of the discrete fit lines.

Further evaluations of the experimental data were conducted at a wavelength of 589 nm due to the availability of refractive indices, as the D-line of the sodium spectrum is usually used for determining the optical properties of materials. The absorption coefficient of PCL was presumed to be 0.0001 µm^−1^, and the considered refractive indices of the used materials were 1.36 for ethanol, 1.33 for PBS, and 1.46 for PCL [23,35,36].

For a simplified and easy-to-use formulation of transmission through nanofibrous scaffolds at distinctive wavelengths, a semi-empirical approach using a regression analysis was adopted using Statistica 10 (StatSoft Inc., Tulsa, OK, USA). In total, the modeling of approximately 250,000 individual experimental data points was performed, and a semi-empirical model, depending on the scaffold properties and surrounding medium, was formulated.

## 3. Results and Discussion

The individual transmission measurements of scaffolds with arbitrary thickness were the basis of the following results. Evaluating the transmission as a function of scaffold thickness for discrete wavelengths opened the possibility to analyze light transmission through electrospun scaffolds and compare devised scaffolds of arbitrary thickness with regard to their transparency.

### 3.1. Individual Transmission Measurements and Resulting Contour Plots

**Figure 4:**
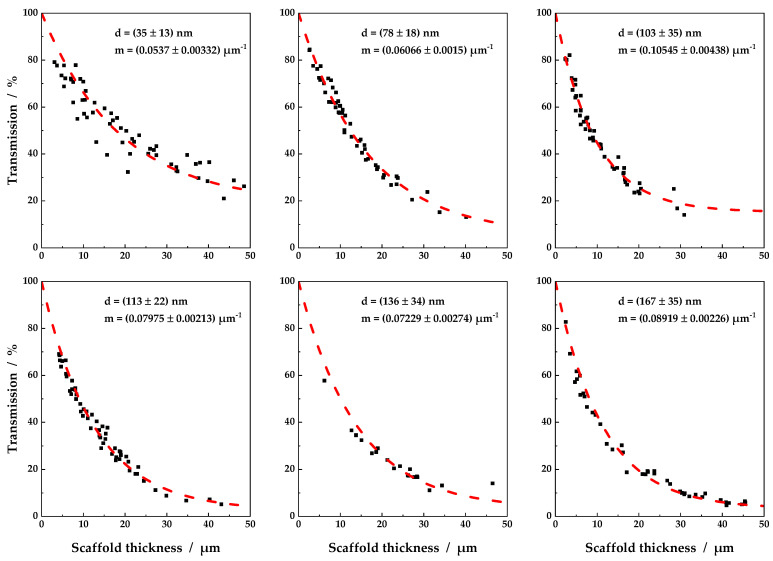
Examples of transmissions as a function of scaffold thickness for a discrete wavelength of 589 nm. Scaffolds were measured in ethanol as the surrounding medium. Fiber diameter increases from top left to bottom right. An exponential decay in light transmission was fitted using Equation (8).

Figure 4 shows schematically the fit functions of all six sample groups for a discrete wavelength of 589 nm. Obviously, as shown in Equation (3), light transmission decreased exponentially with increasing scaffold thickness. Sufficiently high transmission values were only obtained below 5 µm, whereby scaffolds with thinner fiber diameters showed a higher light transmission in general. As displayed, the parameter m from Equation (8) increased with increasing fiber diameter from 0.054 to 0.089, and thus light attenuation. The highest light transmission could therefore be attributed to scaffolds consisting of fibers with a diameter of 35 nm (Figure 4, top left). The parameters of the scaffolds with a fiber diameter of 103 nm and 136 nm slightly diverged from the overall trend. This could be due to insufficient data points in the relevant thickness range, resulting in poor data fitting. Moreover, the broad fiber diameter distribution accounted for insignificant distinguishable median values of the fiber diameters for the samples with a fiber diameter of 103 nm to 136 nm. Nevertheless, it is clear from Figure 4 that with increasing fiber diameter, the coefficient m increased, and transmission of visible light through the scaffolds decreased.

**Figure 5:**
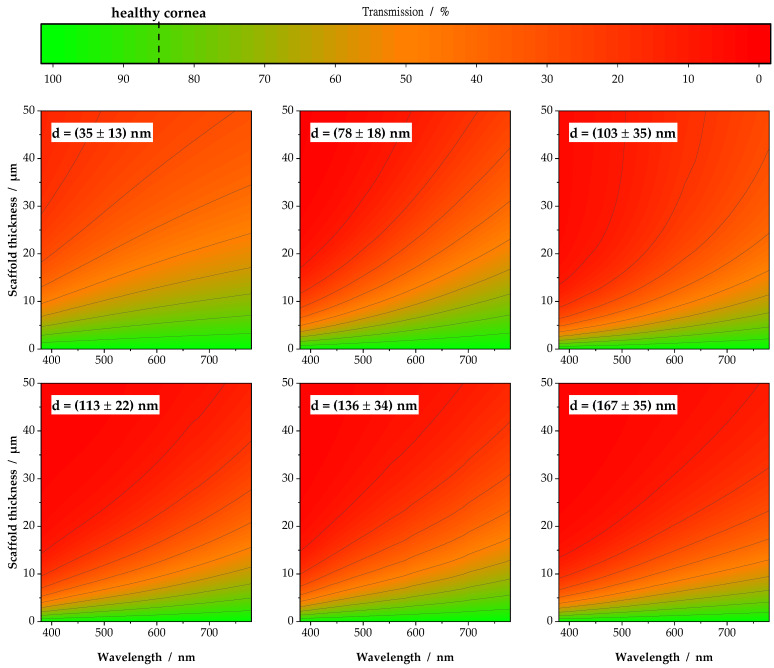
Resulting contour plots from the individual light transmission measurements, as shown in Figure 4. Sufficient light transmission can be observed only for thin scaffolds, as depicted by the color grading, whereby a smaller fiber diameter enhances light transmission through the scaffolds. Fiber diameter increases from top left to bottom right.

The empirical description of light transmission, as shown in Figure 4, was evaluated for discrete wavelengths from 380 nm to 780 nm with an increment of 10 nm. From the combination of fit lines, contour plots were generated, as displayed in Figure 5. The fit lines became vertical lines in Figure 5, with transmission as color grading from red (0% light transmission) to green (100% light transmission). Light transmission >85% characterized a scaffold transparency comparable to that of the human cornea [9]. Again, it became clear that light transmission values above 85% were only accessible for thin scaffolds. With increasing scaffold thickness, light transmission was reduced to values insufficient for all types of scaffolds. The concept presented in this study, using discrete wavelengths and resulting contour plots, as shown in Figure 4 and Figure 5, may serve as a tool to decide on the maximum scaffold thickness for a desired light transmission or vice versa. This represents a novel approach for characterization of scaffolds for corneal tissue engineering. Formerly, for a meaningful comparison, scaffolds of similar thickness had to be produced. Now, for the first time, light transmission through nanofibrous scaffolds can be compared, not only for existing scaffolds but also for scaffolds of arbitrary thickness. Based on the plots in Figure 5, further evaluations of the influence of fiber diameter and enclosing medium on light transmission through electrospun scaffolds were performed.

### 3.2. Influence of Fiber Diameter and Surrounding Medium

**Figure 6:**
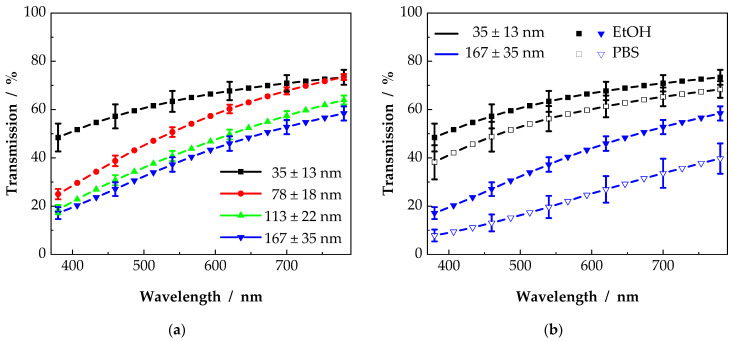
Examples of extracted contour lines from Figure 5. Transmission values were taken for scaffolds with a thickness of 10 µm. Light transmission increases with a decreasing fiber diameter (**a**) as well as with a decreasing ratio of the refractive indices (**b**).

As shown in the previous section, light transmission depends on the scaffold properties. Beside scaffold thickness, fiber diameter is the structuring element. With decreasing fiber diameter, the structure of the scaffolds changed, as the number of fibers per unit volume increases. In Figure 6a, exemplary 10 µm scaffolds from the contour plots of Figure 5 are displayed. It is shown that the overall light transmission increased with decreasing fiber diameter. For a better clarity, scaffolds with 103 nm and 136 nm fiber diameter were left out as, due to the broad fiber diameter distribution of electrospun nanofibers, light transmission values were not significantly different for the scaffolds from 103 nm, 113 nm and 136 nm, as already mentioned before. The highest light transmission was observed for scaffolds consisting of fibers with a diameter of 35 nm. Transmission values up to 66% (at 589 nm) were measured. With increasing fiber diameter, light transmission decreased to 43% (at 589 nm). For all scaffolds, a wavelength-dependent light transmission was observed. This could occur from the decreasing ratio of fiber diameter to wavelength with increasing wavelength. Similar to pure Rayleigh scattering, where the scattered intensity is proportional to λ^−4^, or the thin needle approximation as shown in Equation (7), the influence of scattering is reduced for increasing wavelengths [27].

Electrospun scaffolds usually show a whitish appearance. The big difference in refractive indices between air and polymer leads to strong isotropic reflections and scattering of all wavelengths; hence, the scaffolds appear white. With a decreasing difference in refractive index, reflectance as well as scattering could be minimized, and scaffold transparency improved. In Figure 6b, the transmission data for two different scaffold types is shown. Just like in Figure 6a, light transmission data were taken from the contour plots as horizontal line for a scaffold thickness of 10 µm for scaffolds with 35 nm and 167 nm fiber diameter. Again, light transmission was enhanced with a reduced fiber diameter. Changing the surrounding medium from EtOH to PBS led to a reduced light transmission by 5 to 10 percentage points. The difference in refractive index increased from 0.1 (PCL/EtOH) to 0.13 (PCL/PBS) resulting in an increased light attenuation.

**Figure 7:**
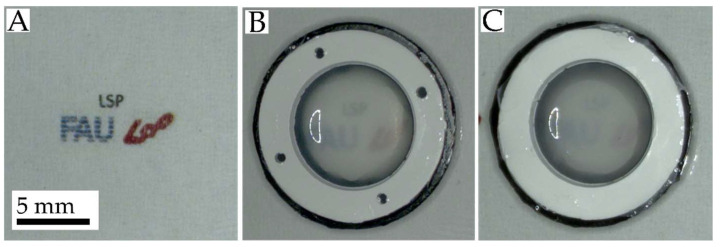
Examples of the transparency of nanofibrous scaffolds: (**A**) blank image as a reference. (**B**) Scaffold consisting of nanofibers with fiber diameters of 35 nm and (**C**) 167 nm. Scaffolds with a thickness close to 10 µm were chosen and moistened in PBS before image recording.

Additionally to UV–vis measurements, differences in light transmission can be observed with optical imaging. Therefore, scaffolds with a thickness close to 10 µm were moistened in PBS and placed onto a reference. The resulting images are displayed in Figure 7. The transparency of the scaffolds, as already indicated in Figure 6, could be classified as insufficient for corneal grafts, though, as shown in Figure 7, the transparency of the scaffold with a mean fiber diameter of 35 nm (B) was closer to that of the reference (A) than the transparency of the scaffold with a fiber diameter of 167 nm (C).

Summarizing the above, it can be concluded that reducing the fiber diameter and matching the refractive indices yield improved light transmission through nanofibrous scaffolds.

### 3.3. Semi-Empirical Description of Light Tranmission

Following the theoretical considerations in the Materials and Methods section, light transmission through the nanofibrous scaffolds depends on scaffold properties such as fiber diameter and scaffold thickness and on material characteristics such as the refractive index. Thus, a semi-empirical description of the experimental transmission data was derived to describe light transmission through nanofibrous scaffolds using regression analysis. With the formulation of scaling laws, precise predictions of the influence of eligible parameters can be made within the experimental accessed range. Neglecting wavelength-dependent variances in the refractive indices and in consistency with the Lambert–Beer law (Equation (3)), the following approach was chosen

(9) $$\ln\frac{-\mathrm{lnT}}{D}{=\alpha }_{0}{+\alpha }_{1}{\mathrm{lnR}+\alpha }_{2}{\mathrm{lnd}+\alpha }_{3}\ln\lambda$$

where R stands for reflectance from equation 5 at a wavelength of 589 nm. The resulting α-values were α_0_ = 1.48, α_1_ = 0.55, α_2_ = 0.60, and α_3_ = 1.68 (d, D and λ in µm). A further simplification based on the consideration of physical reasonable dimensions, led to the improved model

(10) $$\ln\frac{-\mathrm{lnT}}{D}{=\alpha }_{0}{+\alpha }_{1}{\mathrm{lnR}+\alpha }_{2}\ln\frac{d}{\lambda^{3}}$$

with only three adjustable parameters. Now, the resulting α-values were α_0_ = 1.41, α_1_ = 0.55 and α_2_ = 0.57. Taking into considerations the experimental error due to variances in fiber diameter as well as scaffold thickness, α_1_ and α_2_ were set as α_1,2_ = 0.5. Subsequently, the model from Equation (10) could be written as

(11) $$T=\exp\left(-\alpha \sqrt{R\frac{d}{\lambda^{3}}}\;D\right)$$

In this semi-empirical model, α is a dimensionless parameter and was set to α = 2.75. Consequently, the formulation presented in Equation (11) could be written as

(12) $$T=\exp\left(-\;2.75\sqrt{{\left(\frac{n_{1}-{\;n}_{2}}{n_{1}{+n}_{2}}\right)}^{2}\frac{d/µm}{{\left(\lambda /µm\right)}^{3}}}\;D/µm\right)$$

and allowed the prediction of light transmission through nanofibrous scaffolds within typical experimental errors. Accounting for the differences in refractive indices, R was derived from the Fresnel equations for vertical incidence, neglecting multibeam interference [24,25].

**Figure 8:**
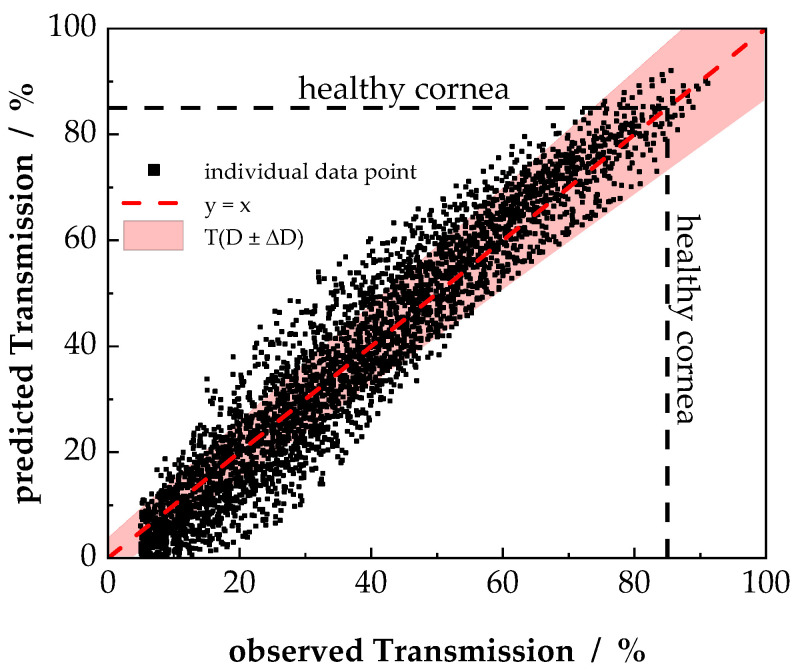
Predicted versus observed transmission of all individual data points. Predicted transmission was calculated using Equation (12). For reasons of clarity, only every 50th data point is shown. The red area corresponds to the error range based on Equations (13) and (21). Transparency of a healthy cornea is indicated at T = 85%.

The predicted transmission data versus the observed transmission data for all six samples groups, measured in two different media within the range of 380 nm to 780 nm, are shown in Figure 8. The data are described with R^2^ = 0.91, suggesting an acceptable accuracy of the model within the experimental data. An estimation of the experimental error was performed utilizing the relative error T_relative_, considering that the dominant experimental uncertainty is attributed to the scattering thickness D.

(13) $$T_{\mathrm{relative}}=\frac{T\left(D\right)-\;T(D\;-\;\Delta D)}{T(D)}\;\approx \;-\sqrt{{\left(\frac{n_{1}-n_{2}}{n_{1}{+n}_{2}}\right)}^{2}\frac{d}{\lambda^{3}}}\;\Delta D\;\approx -{µ(n}_{1}{,\;n}_{2},\;\lambda ,\;d)\Delta D$$

T_relative_ equals approximately −µ ΔD, giving easy access to the expectable accuracy of the predicted transmission data, as µ is defined as µ(n_1_, n_2_, λ, d). In order to estimate the error of the scaffold thickness, the following simple approach was adopted: assuming that a scaffold with total thickness D can be separated in N layers of thickness D_i_, the total thickness can be written as

(14) $$D=\overset{N}{\underset{i=1}{\sum }}D_{i}$$

yielding the error of the total thickness, utilizing error propagation

(15) $$\Delta D=\sqrt{\overset{N}{\underset{i=1}{\sum }}\Delta {D_{i}}^{2}}$$

On the other hand,

(16) $${D=D}_{i}N$$

while all sublayers with thickness D_i_ can be assumed to have the same thickness D_e_

(17) $$D_{i}{=D}_{e}$$

and therefore the same error

(18) $${\Delta D}_{i}{=\Delta D}_{e}$$

From Equation (15), it follows

(19) $$\Delta D=\sqrt{N}{\Delta D}_{e}$$

and utilizing Equation (16), the error can now be estimated with

(20) $$\Delta D=k\sqrt{D}$$

where k is an adjustable parameter. Considering typical values for the scaffold thickness, k yields values of approximately 1 µm. Finally, the experimental error in the measurement of the scaffold thickness can be estimated with

(21) $$\Delta D=1\;µm\;\sqrt{D/µm}$$

With the semi-empirical formulation of light transmission through nanofibrous scaffolds, a novel concept is presented for the design of nanofibrous scaffolds, focusing on the optical properties.

### 3.4. Formulation of the Design Principles

Tissue engineering in the context of ophthalmology mostly deals with the full or lamellar replacement of the cornea. The main part of the cornea, the stroma, consists of highly aligned collagen fibrils [9], which act as scatterers, besides other parts of the stroma like the keratocytes. The collagen fibrils have a diameter around 25 nm and are thus even smaller in diameter than the smallest fibers in this study. Considering them as a blueprint, mimicking the corneal structure would mean the following:

- Reducing the fiber diameter d;
- Reducing the scaffold thickness D;
- Selecting a material with a refractive index similar to that of the human cornea

Meanwhile, the first two points refer to structural properties, while the latter one is purely based on the chosen materials, whereby fiber diameter and matching refractive indices are closely connected, as depicted in Figure 6. Especially with decreasing fiber diameter, light transmission is mainly influenced by the scattering cross section, which again strongly depends on the refractive index of the used material. The ideal material would therefore have a refractive index as close as possible to the refractive index of the human cornea with n_cornea_ = 1.376 [9].

**Figure 9:**
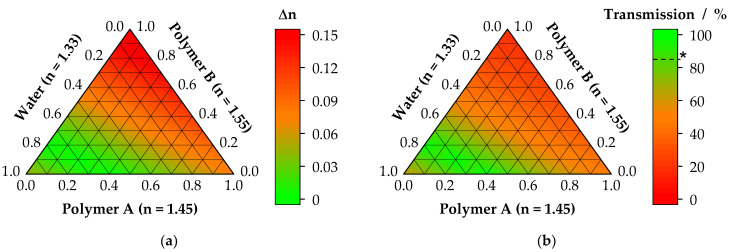
Example for a mixture of two polymers and different water uptake after swelling. From the refractive indices of the single materials, the overall refractive index as well as the difference with respect to the refractive index of the cornea can be calculated (**a**). Using Equation (11), the resulting light transmission through such hypothetic scaffolds can be estimated (**b**). Scaffold was defined to be 10 µm thick, consisting of fibers with a fiber diameter, after swelling, of 100 nm. Transmission is shown at 589 nm. Asterisk (*) indicates corneal transparency corresponding to T_cornea_ > 85%.

As most of the commonly used polymers for tissue engineering possess a refractive index of approximately 1.50, it becomes evident that for corneal tissue engineering, the use of pure polymers will result in insufficient light transmission. Therefore, we suggest blending these polymers with foremost hygroscopic polymers such as peptides or polysaccharides or even using hygroscopic polymers themself as fibers for the scaffold. The key to an improved light transmission lies in the incorporation of water (n = 1.33) in the polymeric fiber matrix. With a sufficient amount of water uptake, the resulting refractive index of the blend fibers can be approximated using the Gladstone–Dale equation [37], which holds for Δn_i_ < 0.2. Thus, an n_total_ can be calculated as

(22) $$n_{\mathrm{total}}=\sum^{\;}n_{i}v_{i}$$

where n_i_ represents the refractive indices, and v_i_ the volume fractions of the individual components. For the sum of the volume fractions applies ∑v_i_ = 1. With the estimation of a hypothetic refractive index for varying blend compositions, the transmission can be predicted. With this approach, a preselection of suitable polymers and polymer blends can be achieved, and basic design principles can be formulated. In Figure 9, a hypothetical example using this approach is shown. The blending of polymer A with polymer B with refractive indices of n_A_ = 1.45 and n_B_ = 1.55 with different ratios requires a defined amount of water uptake to minimize the difference in refractive indeces between the ternary blend and the natural cornea. As a visual result, the ternary contour plot of Δn is shown in Figure 9a. From this on, using Equation (11), the transmission could be calculated. In Figure 9b, the transmission for a scaffold in equilibrium swelling state with 10 µm thickness, 100 nm fiber diameter, and an experimental wavelength of 589 nm is shown.

The resulting proportion of polymer A, B, and water refers to the steady state, where the swelling reached its equilibrium value. While the ratio of polymer A to B can be adjusted as desired, water uptake is mainly dependent on the hygroscopic behavior of the blend. In the case of Figure 9, a very low amount of the components A and B in the range from 0.2 to 0.5 and 0 to 0.3 should be used, while a high water uptake is required, leading to a final water content of 0.7 to 0.8 (70–80%). Such blends will show high light transmission values over 85%, qualifying for corneal grafts.

Table 2 provides a brief overview of various eligible blend polymers. For most polymers, swelling, and thus water uptake, are highly dependent on the degree of crosslinking, the crosslinking agent, and the molar mass. It must be pointed out that hygroscopic polymers require chemical or physical crosslinking, otherwise the fibers would lose their mechanical strength due to the water uptake or will be dissolved in the worst case. In the case of polymer blends, water uptake is related to the blend polymer and relative amounts of the matrix and blend polymer. The approach presented in this study can be used in all areas of biomaterials like bio-printing or tissue engineering, where transparency of the graft is of interest.

## 4. Conclusions

The transmission of light in the visible spectrum from 380 nm to 780 nm is an important characteristic of future transplants in corneal tissue engineering. Should the patient experience a direct improvement after surgery, transparent grafts must be produced. With the emerging interest in electrospun scaffolds for corneal tissue engineering, transparency has to be equally important to biocompatibility and mechanical strength. In the literature, graft transparency is only examined as a side aspect of graft evaluation, and in most publications only exemplary grafts are shown. In this study, a detailed analysis of light transmission through nanofibrous PCL scaffolds was performed. By varying fiber diameter and surrounding medium, material and structural properties could be separated. For enhanced transparency of nanofibrous scaffolds, thin fibers and matching refractive indices should be used. Moreover, a novel, simple model is provided to describe the light transmission of nanofibrous scaffolds and its experimental validation by a huge amount of data. Finally, from the general conclusions, design principles were formulated to promote further research in the field of corneal tissue engineering

## Figures and Tables

**Table 1:** Parameters for the electrospinning of PCL scaffolds from spinning solutions with varying concentrations from 5 g/100 mL to 16 g/100 mL and resulting fiber diameters.

Concentration (g/100 mL)	Distance (cm)	High Voltage (kV)	Flow Rate (mL/h)	Fiber Diameter (nm)
5	15	15	0.2	35 ± 13
8	10	10	0.15	78 ± 18
10	15	15	0.1	103 ± 35
12	17	15	0.2	113 ± 22
14	15	15	0.25	136 ± 34
16	15	15	0.2	167 ± 35

**Table 2:** Examples of eligible hygroscopic polymers for tissue engineering of transparent tissues, e.g., corneal transplant. The table does not lay any claim to completeness.

Polymer	Swelling ^1^/%	Reference
gelatin	150–300	[38,39]
zein	5–35	[40,41]
alginate	50–150	[42,43]
chitosan	100–900%	[42,44,45]

^1^ Depending on crosslinking, crosslinking agent, and/or blend polymer and content.

## Data Availability

The data presented in this study are available on reasonable request from the corresponding author.

## References

[1] Bosworth L.A., Downes S. (2011). Electrospinning for Tissue Regeneration.

[2] Küng F., Schubert D.W., Stafiej P., Kruse F.E., Fuchsluger T.A. (2016). A novel suture retention test for scaffold strength characterization in ophthalmology. Mater. Sci. Eng. C Mater. Biol. Appl..

[3] Küng F., Schubert D.W., Stafiej P., Kruse F.E., Fuchsluger T.A. (2017). Influence of operating parameters on the suture retention test for scaffolds in ophthalmology. Mater. Sci. Eng. C Mater. Biol. Appl..

[4] Stafiej P., Küng F., Kruse F.E., Schubert D.W., Fuchsluger T. (2018). Mechanical and Optical Properties of PCL Nanofiber Reinforced Alginate Hydrogels for Application in Corneal Wound Healing. Biomater. Med Appl..

[5] Stafiej P., Küng F., Thieme D., Czugala M., Kruse F.E., Schubert D.W., Fuchsluger T.A. (2017). Adhesion and metabolic activity of human corneal cells on PCL based nanofiber matrices. Mater. Sci. Eng. C Mater. Biol. Appl..

[6] Kim J.I., Kim J.Y., Park C.H. (2018). Fabrication of transparent hemispherical 3D nanofibrous scaffolds with radially aligned patterns via a novel electrospinning method. Sci. Rep..

[7] Kruse M., Walter P., Bauer B., Rütten S., Schaefer K., Plange N., Gries T., Jockenhoevel S., Fuest M. (2018). Electro-spun Membranes as Scaffolds for Human Corneal Endothelial Cells. Curr. Eye Res..

[8] Himmler M., Garreis F., Paulsen F., Schubert D.W., Fuchsluger T.A. (2021). Optimization of polycaprolactone—Based nanofiber matrices for the cultivation of corneal endothelial cells. Sci. Rep..

[9] Levin L.A., Kaufman P.L. (2011). Adler’s Physiology of the Eye: Clinical Application.

[10] Yoeruek E., Bayyoud T., Maurus C., Hofmann J., Spitzer M.S., Bartz-Schmidt K.-U., Szurman P. (2012). Decellularization of porcine corneas and repopulation with human corneal cells for tissue-engineered xenografts. Acta Ophthalmol..

[11] Lynch A.P., Ahearne M. (2013). Strategies for developing decellularized corneal scaffolds. Exp. Eye Res..

[12] Ponce Márquez S., Martínez V.S., McIntosh Ambrose W., Wang J., Gantxegui N.G., Schein O., Elisseeff J. (2009). Decellularization of bovine corneas for tissue engineering applications. Acta Biomater..

[13] Salehi S., Czugala M., Stafiej P., Fathi M., Bahners T., Gutmann J.S., Singer B.B., Fuchsluger T.A. (2017). Poly (glycerol sebacate)-poly (ε-caprolactone) blend nanofibrous scaffold as intrinsic bio-and immunocompatible system for corneal repair. Acta Biomater..

[14] Ozcelik B., Brown K.D., Blencowe A., Daniell M., Stevens G.W., Qiao G.G. (2013). Ultrathin chitosan-poly (ethylene glycol) hydrogel films for corneal tissue engineering. Acta Biomater..

[15] Ozcelik B., Brown K.D., Blencowe A., Ladewig K., Stevens G.W., Scheerlinck J.-P.Y., Abberton K., Daniell M., Qiao G.G. (2014). Biodegradable and biocompatible poly (ethylene glycol)-based hydrogel films for the regeneration of corneal endothelium. Adv. Healthc. Mater..

[16] Tummala G.K., Lopes V.R., Mihranyan A., Ferraz N. (2019). Biocompatibility of Nanocellulose-Reinforced PVA Hydrogel with Human Corneal Epithelial Cells for Ophthalmic Applications. J. Funct. Biomater..

[17] Kong B., Chen Y., Liu R., Liu X., Liu C., Shao Z., Xiong L., Liu X., Sun W., Mi S. (2020). Fiber reinforced GelMA hydrogel to induce the regeneration of corneal stroma. Nat. Commun..

[18] Tonsomboon K., Oyen M.L. (2013). Composite electrospun gelatin fiber-alginate gel scaffolds for mechanically robust tissue engineered cornea. J. Mech. Behav. Biomed. Mater..

[19] Chen Y.-L., Chang Y.-H., Huang J.-L., Chen I., Kuo C. (2012). Light Scattering and Enhanced Photoactivities of Electrospun Titania Nanofibers. J. Phys. Chem. C.

[20] Wu H., Hu L., Rowell M.W., Kong D., Cha J.J., McDonough J.R., Zhu J., Yang Y., McGehee M.D., Cui Y. (2010). Electrospun metal nanofiber webs as high-performance transparent electrode. Nano Lett..

[21] Khudiyev T., Huseyinoglu E., Bayindir M. (2014). Non-resonant Mie scattering: Emergent optical properties of core-shell polymer nanowires. Sci. Rep..

[22] Mahmoud Salehi A.O., Heidari Keshel S., Sefat F., Tayebi L. (2021). Use of polycaprolactone in corneal tissue engineering: A review. Mater. Today Commun..

[23] Park C., Woon Choi H., Lee C.H., Lannutti J.J., Farson D.F. (2014). Optical scattering in electrospun poly (ε-caprolactone) tissue scaffolds. J. Laser Appl..

[24] Born M., Wolf E., Bhatia A.B. (2016). Principles of Optics: Electromagnetic Theory of Propagation, Interference and Diffraction of Light.

[25] Bennett J.M., Bass M., Mahajan V.N. (2010). Handbook of Optics: Volume I—Geometrical and Physical Optics, Polarized Light, Components and Instruments.

[26] Rayleigh X. (1881). On the electromagnetic theory of light. Lond. Edinb. Dublin Philos. Mag. J. Sci..

[27] van de Hulst H.C. (2012). Light Scattering by Small Particles.

[28] Hart R.W., Farrell R.A. (1969). Light scattering in the cornea. J. Opt. Soc. Am..

[29] Cox J.L., Farrell R.A., Hart R.W., Langham M.E. (1970). The transparency of the mammalian cornea. J. Physiol..

[30] Freund D.E., McCally R.L., Farrell R.A. (1986). Direct summation of fields for light scattering by fibrils with applications to normal corneas. Appl. Opt..

[31] Farrell R.A., McCally R.L., Albert D.M., Jakobiec F.A. (2000). Corneal Transparency. Principles and Practice of Ophthalmology.

[32] Freund D.E., McCally R.L., Farrell R.A., Cristol S.M., L’Hernault N.L., Edelhauser H.F. (1995). Ultrastructure in anterior and posterior stroma of perfused human and rabbit corneas. Relation to transparency. Invest. Ophthalmol. Vis. Sci..

[33] Wendorff J.H., Agarwal S., Greiner A. (2012). Electrospinning: Materials, Processing and Applications.

[34] Schubert D.W. (2019). Revealing Novel Power Laws and Quantization in Electrospinning Considering Jet Splitting—Toward Predicting Fiber Diameter and Its Distribution. Macromol. Theory Simul..

[35] Rheims J., Köser J., Wriedt T. (1997). Refractive-index measurements in the near-IR using an Abbe refractometer. Meas. Sci. Technol..

[36] Van Hoang T., Stępniewski G., Czarnecka K.H., Kasztelanic R., van Long C., Xuan K.D., Shao L., Śmietana M., Buczyński R. (2019). Optical Properties of Buffers and Cell Culture Media for Optofluidic and Sensing Applications. Appl. Sci..

[37] Gladstone J.H., Dale P.T. (1863). XIV. Researches on the refraction, dispersion, and sensitiveness of liquids. Phil. Trans. R. Soc..

[38] Bigi A., Cojazzi G., Panzavolta S., Roveri N., Rubini K. (2002). Stabilization of gelatin films by crosslinking with genipin. Biomaterials.

[39] Zhuang C., Tao F., Cui Y. (2015). Anti-degradation gelatin films crosslinked by active ester based on cellulose. RSC Adv..

[40] Han Y.-L., Xu Q., Lu Z.-Q., Wang J.-Y. (2014). Preparation of transparent zein films for cell culture applications. Colloids Surf. B Biointerfaces.

[41] Budi Santosa F.X., Padua G.W. (1999). Tensile properties and water absorption of zein sheets plasticized with oleic and linoleic acids. J. Agric. Food Chem..

[42] Remuñán-López C., Bodmeier R. (1997). Mechanical, water uptake and permeability properties of crosslinked chitosan glutamate and alginate films. J. Control. Release.

[43] Jejurikar A., Seow X.T., Lawrie G., Martin D., Jayakrishnan A., Grøndahl L. (2012). Degradable alginate hydrogels crosslinked by the macromolecular crosslinker alginate dialdehyde. J. Mater. Chem..

[44] Khalid M.N., Agnely F., Yagoubi N., Grossiord J.L., Couarraze G. (2002). Water state characterization, swelling behavior, thermal and mechanical properties of chitosan based networks. Eur. J. Pharm. Sci..

[45] Correlo V.M., Pinho E.D., Pashkuleva I., Bhattacharya M., Neves N.M., Reis R.L. (2007). Water absorption and degradation characteristics of chitosan-based polyesters and hydroxyapatite composites. Macromol. Biosci..

